# Analytical evaluation of TriVerity, a rapid diagnostic and prognostic host gene expression test performed on the Myrna instrument using RT-LAMP

**DOI:** 10.1128/jcm.00352-25

**Published:** 2025-08-27

**Authors:** Claudia Figueiredo-Pereira, Paul Fleming, Mikaela Nicole Alganes, Ran Bi, Ana Mafalda Cavaleiro, Diogo Cruz, Carlota Cunha-Matos, Margarita Davalos-Arias, Dana Farkas, Yehudit Hasin, Kevin Hu, Christos Kampouridis, Ragheb El Khaja, Graciano Leal, Jingyi Lu, Rita Madeira, Michael Mayhew, Breana McBryde, Daniela C. Oliveira, Anna Passernig, Davion Pendleton, Elizabeth Popp, Shailee Rasania, Cristina Rebelo, Ana Santiago, Joshua R. Shak, Vera P. Silva, Ambika Srinath, Timothy E. Sweeney, Rodrigo Vieira, Thang Vu, Chris Wilson, Boris Zybin, Natalie N. Whitfield, Oliver Liesenfeld, Hjalmar R. Bouma, Richard E. Rothman, Edward A. Michelson, Joao Fonseca

**Affiliations:** 1 Inflammatix, Inc. 523007 , Sunnyvale, California, USA; 2 Department of Acute Medicine, University of Groningen 3647, Groningen, the Netherlands; 3 Adult Emergency Department, Johns Hopkins Hospital 588543, Baltimore, Maryland, USA; 4 Department of Internal Medicine, University of Groningen 3647, Groningen, the Netherlands; 5 Department of Clinical Pharmacy & Pharmacology, University Medical Center Groningen, University of Groningen 3647, Groningen, the Netherlands; 6 Department of Emergency Medicine, Texas Tech University 6177, El Paso, Texas, USA; Cleveland Clinic, Cleveland, Ohio, USA

**Keywords:** sepsis, host response, acute infection, diagnostics, TriVerity, Myrna

## Abstract

We evaluated the analytical performance of the TriVerity test system, a benchtop system for rapid measurement and interpretation of host messenger RNAs (mRNAs) measured quantitatively from PAXgene Blood RNA specimens using the TriVerity cartridge on the Myrna instrument. In approximately 30 minutes, 3 scores are generated from 29 host mRNAs for the likelihood of a bacterial infection, a viral infection, and illness severity (7-day need for ICU-level care), each falling into 1 of 5 interpretation bands (very low, low, moderate, high, and very high). Reproducibility, precision, limit of detection, linearity, interference, and sample and cartridge stability were determined per Clinical Laboratory Standards Institute standards. An operator survey assessed TriVerity’s ease of use. Reproducibility demonstrated SDs meeting the acceptance criteria of <5.5 score units. The lowest concentration at which 100% of replicates showed measurable amplification was 1 × 10^6^ cp/mL of *in vitro* RNA transcripts; the limit of quantification and detection were equivalent to blood containing 500 leukocytes/µL. Interference was not observed for 17 potential interferents. The cartridge was stable at room temperature (RT) for 9 and 12 months using accelerated testing at 37°C. TriVerity results were equivalent using fresh and frozen PAXgene Blood RNA samples. All operators agreed or strongly agreed that the TriVerity test system was easy to use. In summary, the analytical performance presented here, along with diagnostic accuracy established in the SEPSIS-SHIELD trial, demonstrates that the TriVerity system is reliable and user friendly, assisting clinicians in managing patients with suspected acute infections and sepsis in acute care settings.

**IMPORTANCE:**

The prompt diagnosis of acute infections and sepsis is critical for better patient outcomes. We introduce a groundbreaking messenger RNA-based test, the first of its kind, designed to diagnose the presence of infection and predict illness severity in adult patients with suspected acute infections or sepsis. Several key findings regarding the accuracy and robustness of the test system are presented, which will be relevant to laboratories or acute care settings implementing the test for patient care. Furthermore, these findings may assist clinical researchers in developing analytical trial protocols aimed at the combined evaluation of RNA-based multi-marker tests with both diagnostic and prognostic test characteristics.

## INTRODUCTION

Patients often present to the emergency department (ED) with suspected infection and/or sepsis; however, alternative diagnoses are possible (1). Conversely, patients with infections may present with undifferentiated complaints and without clear signs of infection, such as fever. Sepsis, defined as life-threatening organ dysfunction caused by a dysregulated host response to an infection (2), requires rapid administration of antimicrobials and fluids (3), as the risk of death from severe sepsis increases by 7%–8% with every hour of delay in beginning treatment (4, 5). The diagnosis of sepsis can be nuanced and frequently missed or delayed, with potentially deadly consequences. Due to the challenges in diagnosis and the need for prompt treatment, many patients presenting with suspected sepsis but ultimately having alternative diagnoses receive unnecessary antimicrobials and hospitalization, which is costly, potentially harmful, and contributes to antibiotic resistance (6).

The two main components of sepsis treatment, anti-infective therapy (e.g., antimicrobials and surgical source control) and supportive therapy (e.g., mechanical ventilation, vasopressor medications, etc.), underscore the need for assessing a patient with suspected infection along two “axes”: (i) infection presence and (ii) illness severity/all-cause likelihood of deterioration (7, 8). Diagnostic tests used in clinical practice today do not reliably distinguish bacterial or viral infection from non-infectious etiologies or predict a patient’s likelihood of decompensating or illness severity (3, 7, 9). For example, while protein-based biomarkers such as C-reactive protein (CRP) and procalcitonin or direct pathogen detection can inform the presence of infection, they often lack the necessary accuracy or speed to enable a physician to accurately identify infections and distinguish bacterial from viral infections at initial presentation (10, 11). Regardless of a patient’s infection status, anticipating the likelihood of decompensation can be even more challenging. Lactate levels and clinical scores (e.g., sequential organ failure assessment [SOFA] and quick SOFA [qSOFA]) have limited accuracy for predicting decompensation in broader populations, particularly among patients who screen negative for systemic inflammatory response syndrome or qSOFA criteria (12).

The TriVerity test system consists of the Myrna instrument and the TriVerity cartridge. It aims to address the unmet medical need of diagnostic and prognostic uncertainty in “gray zone” patients in the ED by measuring the host’s blood messenger RNA (mRNA) levels to diagnose both the presence of infection and the severity of illness. The accuracy of host gene amplification combined with machine learning algorithms used by TriVerity was previously demonstrated in observational studies (12–16); TriVerity’s clinical performance on the Myrna instrument was recently reported in the SEPSIS-SHIELD study (NCT04094818) (17).

Here, we describe the validation of the TriVerity test system using analytical studies designed in alignment with Clinical Laboratory Standards Institute (CLSI) guidelines and Food and Drug Administration (FDA) feedback. Along with an operator survey, these results demonstrate the accuracy, robustness, and ease of use of the TriVerity test system.

## MATERIALS AND METHODS

### Study design

The planning, conduct, and statistical analysis of the analytical studies were guided by CLSI guidelines, presubmission interaction with the FDA, and interactive sprint sessions conducted via the FDA’s Breakthrough Device Designation program. FDA clearance for the TriVerity test was obtained on 10 January 2025 (K241676).

### Testing sites

Most analytical studies were performed at the Inflammatix Clinical Laboratory (Sunnyvale, CA, USA). The multi-site reproducibility and precision studies were conducted at Inflammatix, the Johns Hopkins Hospital Emergency Medicine Research Laboratory (MD, USA), and Texas Tech University Health Sciences Center Emergency Medicine Research Laboratories (TX, USA). The fresh-frozen equivalency study was performed at Groningen University Medical Center (Groningen, The Netherlands).

### Patient sample collection

Subject specimen collection was performed in accordance with the International Conference on Harmonization-Good Clinical Practice guidelines and followed local regulatory requirements. Written consent was obtained from each participant using the Institutional Review Board (IRB)/Independent Ethics Committee-approved Informed Consent Form. Whole blood (2.5 mL) was collected into PAXgene Blood RNA tubes (PreAnalytiX, Hombrechtikon, Switzerland).

For the analytical precision study, 143 samples were selected from subjects enrolled in the SEPSIS-SHIELD trial (NCT04094818) with suspected acute infection and/or suspected sepsis, with at least 20 samples from each of the 15 interpretation bands (5 each per bacterial, viral, or severity). For the fresh-frozen equivalency study, we included samples from 60 subjects enrolled in the Acutelines study at University Medical Center Groningen, Department of Acute Care, with a suspected bacterial or viral infection (18). The TriVerity test was performed locally. The study was approved by the UMCG IRB (PaNaMa number 16775).

### TriVerity test system

The Myrna instrument is a fully automated benchtop platform that performs nucleic acid isolation and multiplexed, quantitative reverse transcription loop-mediated isothermal amplification (qRT-LAMP) on cartridge consumables. The instrument has a small footprint with dimensions of 20.00 inches (510 mm) in length × 11.50 inches (292 mm) in width × 10.50 inches (267 mm) in height. The Myrna instrument is stackable and requires no calibration. The Myrna instrument’s user interface guides the operator through performing a test. The system can be configured to transfer results automatically to a laboratory information system (LIS) or electronic health record (EHR) via ethernet (port on the back of the instrument) or through a wireless connection.

**Fig 1:**
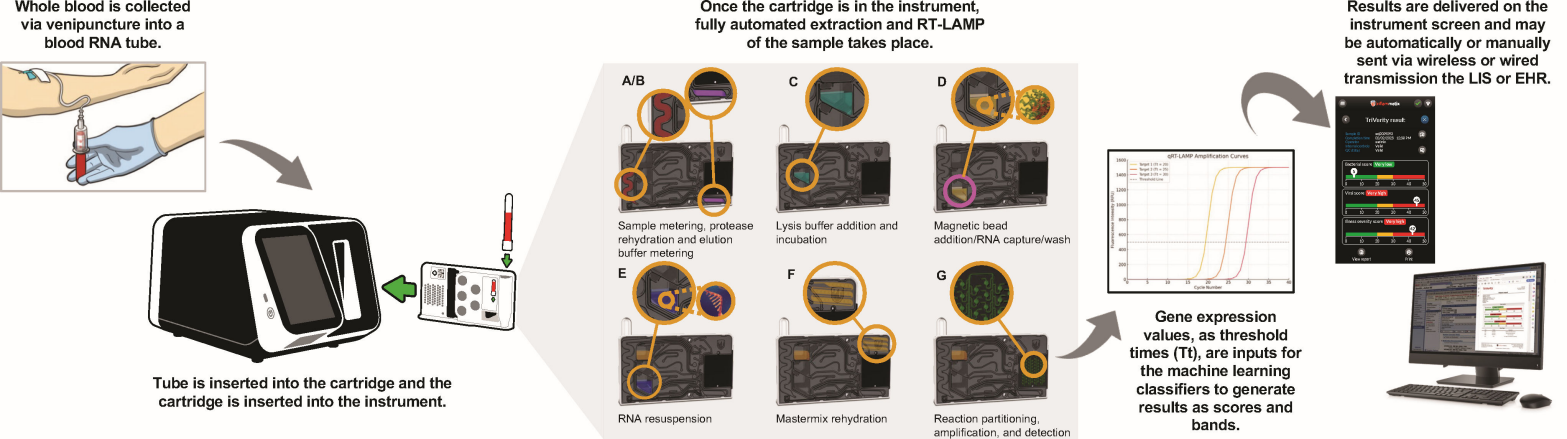
Schematic overview of the TriVerity test system workflow. LIS, laboratory information system; EHR, electronic health record.

The TriVerity single-use test cartridge was developed for the Myrna instrument (Fig. 1). Upon insertion of the cartridge into the instrument, approximately 500 µL of the PAXgene blood sample is drawn into the cartridge, where magnetic bead-based nucleic acid isolation occurs. Samples collected in the incorrect tube type yield a test error, rather than providing potentially erroneous results. The RT-LAMP mastermix is rehydrated and combined with isolated nucleic acid, which is then partitioned into 68 discrete reactions, each containing a unique primer set. The Myrna instrument provides thermal control for isothermal LAMP and includes a single-color optics system for real-time fluorescence monitoring to yield results in approximately 30 minutes.

Gene amplification values, as threshold times (Tt), are generated for 29 informative genes, 3 housekeeping genes, and 2 internal controls. Informative genes (Table 1) have been described previously (8, 19, 20). Housekeeping genes normalize input RNA, while process (internal) controls ensure RNA extraction and RT-LAMP steps are working correctly, including the detection of inhibition. The 29 host response genes provide input vectors for the TriVerity classifiers Inflammatix Bacterial Viral Noninfected-4 (IMX-BVN-4) and Severity-4 (IMX-SEV-4) to estimate the likelihood of a bacterial infection, the likelihood of a viral infection, and the risk of severe outcomes within 7 days, i.e., the need for vasopressors, mechanical ventilation, and/or renal replacement therapy (17). Both classifiers interpret mRNA gene amplification, Tts, to produce probabilities of class assignment, which are then converted to scores (19). TriVerity yields three scores: (i) bacterial score, (ii) viral score, and (iii) illness severity score. Each score (range 0–50) falls into an interpretation band: very high (41–50), high (31–40), moderate (21–30), low (11–20), and very low (0–10).

**TABLE 1:** Informative target host response genes, housekeeping genes, and process controls used in the TriVerity test

Target genes of the TriVerity test		
Host response genes		
ANKRD22	ARG1	BATF
C3AR1	CD163	CEACAM1
CLEC5A	CTSL1	DEFA4
HERC5	HLA-DMB	IFI27
IFI44	IFI44L	IL18R1
IL1R2	ISG15	JUP
KCNJ2	LY86	OASL
OLFM4	PSMB9	RSAD2
S100A12	TDRD9	TGFBI
XAF1	ZDHHC19	
Housekeeping genes		
KPNA6	RREB1	YWHAB
Process (internal) control genes		
ERCC17	ERCC59	

### External controls

Calibration of the Myrna instrument is not required. While not a requirement of the TriVerity system, two external controls can be used to verify the proper functioning of the Myrna instrument and TriVerity cartridges with each new lot of cartridges. External control level 1 generates a very high bacterial, very low viral, and very high illness severity score, whereas external control level 2 generates a very low bacterial, very high viral, and very low severity score. The Myrna instrument displays a valid or invalid status after running external controls.

### Reproducibility

The reproducibility study was performed according to the CLSI EP05-A3 guidelines (21) at one internal and two external sites. Testing occurred twice daily over five non-consecutive days on three instruments at each site. Each of the two daily runs was performed by a different operator. Three replicates each of four contrived samples (blood sample matrix supplemented with *in vitro* transcribed RNA) spanning the range of results in the TriVerity test were processed. Expected scores for contrived samples were as follows: sample A, high bacterial, moderate viral, and high severity; sample B: very high bacterial, very low viral, and very high severity; sample C: very low bacterial, very high viral, and very low severity; sample D: very low bacterial, very low viral, and very low severity. Instrument and cartridge parameters to determine lot-to-lot reproducibility are shown in Table S1. Contrived samples were prepared at Inflammatix and shipped on dry ice to testing sites. The acceptance criterion was an SD of less than 5.5 score units for each result (bacterial, viral, and severity) and less than 2.5% of scores falling into a non-adjacent interpretation band. This criterion was established to minimize the likelihood of scores falling into non-adjacent bands, which could impact clinical decision-making.

### Precision

To characterize the precision of the system, 143 clinical patient samples covering all TriVerity test interpretation bands, with at least 15 results in every interpretation band, were run in duplicate where each duplicate used the same instrument and the same cartridge lot. The study was performed over 5 days using 3 different cartridge lots and 12 different instruments. SD was calculated for each score (bacterial/viral/ severity) and for each interpretation band (15 bands) excluding outliers per Grubb’s outlier analysis technique. Similar to the reproducibility study, the acceptance criteria for each band on each axis were set at SD <5.5 score units.

### Lot-to-lot reproducibility

Lot-to-lot reproducibility was assessed using a consistent measurement procedure, instrument, operator, operating conditions, and location to obtain replicate measurements of the same sample using three different cartridge lots (Table S1). The study was executed over 3 days using one instrument per sample level, one operator, four contrived samples, and cartridges from three different lots. For each of the four contrived sample levels, a single Myrna instrument was used to run sample replicates in cartridges with alternating runs for each lot, resulting in six replicates per lot/sample level. This design was applied to all four contrived sample levels to characterize the reproducibility of the different score bands across different cartridge lots. Like the reproducibility study, the acceptance criteria for each band on each axis were set at SD <5.5 score units. Analysis of data obtained in this study was performed using a nested ANOVA model with cartridge lot nesting with the restricted maximum likelihood (REML) criterion as the model objective and utilizing Grubbs’ outlier analysis.

### Analytical sensitivity

Analytical sensitivity was assessed per the CLSI: EP17-A2 standard (22). Sample concentrations ranged from 1 × 10^5^ to 1 × 10^6^ copies/mL of *in vitro* RNA transcripts, in accordance with observed concentrations of target RNA in clinical samples. Samples were prepared according to Table S4. Each subsequent concentration was prepared by serially diluting the previous concentration in RNA-stabilizing solution. The analytical sensitivity protocol was executed by a single operator, over 3 days, using 3 instruments, testing 20 replicates per concentration. The testing was performed in descending order, from the higher concentrated sample to the lowest. The analytical sensitivity was determined to be the lowest concentration at which 95% (19/20) of samples for each individual gene tested demonstrated measurable amplification.

### Linearity

Linearity was assessed using target concentrations from 5 × 10^5^ to 5 × 10^9^ copies/mL of *in vitro* RNA transcripts, in accordance with observed concentrations of target RNA in clinical samples. Linearity samples were prepared using pure *in vitro* transcribed RNA sequences corresponding to each of the 32 mRNAs spiked into an RNA stabilizing solution (Table S4). Each subsequent concentration was prepared by serially diluting the previous concentration in RNA stabilization buffer. Testing was performed by a single operator, on a single day, using a single instrument, testing two replicates per concentration. The testing was performed in descending order, from the highest concentrated sample to the lowest. Linearity was determined to be the range of concentration within 90% confidence limits and a 5% allowable deviation from linearity (ADL).

### Limit of quantitation

The limit of quantitation (LoQ) is the lower limit of analyte concentration that can be reliably measured, such that the total error meets a predefined total error accuracy goal. Due to the multiplexed nature of the assay, there were neither specimens available with low concentrations for all analytes nor a practical study design possible to assess a combination of clinical samples providing low concentrations for all analytes. The approach was to create clinical pools that span the spectrum of possible scores and dilute each pool until the total error met a predefined total error accuracy goal. Two clinical pools were prepared: (i) pool A (low bacterial/high viral/low severity) and (ii) pool B (high bacterial/low viral/high severity). Pools were created by combining individual clinical samples stored in PAXgene Blood RNA tubes, with white blood cell (WBC) counts of 1,995 cells/µL for pool A and 1,296 cells/µL for pool B. Two lots of TriVerity test cartridges were tested across multiple concentrations, days (>3 days), instruments, and sample types, totaling 190 runs. Each sample concentration was prepared according to Table S2 by serially diluting the clinical pools in leukocyte-reduced blood.

Testing was performed in descending order, from the highest concentrated sample (1,500–2,000 cell/uL) to the lowest (125 cells/uL) (Table S2). For each tested concentration, a *t*-test was performed to determine if there was a statistically meaningful shift in the mean score (for all three scores) between the base concentration and the testing concentration. If there was a statistically significant shift, the size of the shift was determined. For each concentration, the percentage of replicates that gave a valid result was calculated. The LoQ for each sample pool was defined as the lowest WBC concentration at which 95% (19/20) of the measured replicates provided a score, and the SD was less than 5.50 score units for all three score types. The overall LoQ was defined as the higher of the LoQs calculated for each sample pool.

### Interference

Each interfering substance (selected based on FDA requirements) was spiked into four tubes of both panels B and C and tested with two different cartridge lots, resulting in eight tests per interfering substance. The formulation of panels B and C was the same as that described for the reproducibility studies; however, multiple lots of each panel were produced for use across several studies. The interfering substances and their concentrations are detailed in Table S3. Acceptance criteria were no significant bias or imprecision on spiked contrived samples when compared to the unspiked control condition, measured as the mean drift between control and test samples <5.50 for each result score and results for the test group within ±1 score band of the control.

### Cross-reactivity

Non-specific amplification of genomic DNA (gDNA) was evaluated by spiking 20 ng/µL of gDNA into RNA stabilization buffer, resulting in a total of 20 runs. Separately, cross-reactivity was assessed by spiking 10 ng/µL of gDNA into two levels of contrived (spiked) samples with previously characterized score results and comparing them with unspiked samples, with six test and six control runs. No-template amplification was evaluated by running blank RNA stabilization buffer samples, totaling 20 runs. Acceptance criteria were no measurable amplification signal in any of the markers from 20 ng/µL human genomic DNA template for at least 95% (19/20) of runs. Test scores should not be impacted by gDNA spiked in at 10 ng/µL for the two contrived sample types, and the mean drift between control and result samples should be <5.50 for each result score and SD of test samples <5.50 for each result score. No-template samples should not provide amplification signal in any of the markers for at least 95% (19/20) of the runs.

### Cartridge stability

Long-term stability was evaluated using real-time stability data at 6 and 9 months and 12 months of accelerated stability data. Stability testing was conducted using two contrived samples (panel members B and C) at two storage temperatures (15°C and 30°C) and an accelerated condition (37°C). For real-time stability, samples were tested at 3, 6, and 9 months, while accelerated stability was assessed at weeks 9, 18, 26, and 34. At each time point, three cartridge lots were tested, with duplicate runs per sample panel member, totaling six runs per sample. Stability was determined using linear regression analysis of score vs. time, with a maximum allowable drift of 5.5 score units between stability time points (i.e., 0 vs 6 or 9 months). If the slope *P*-value was ≥0.05, stability was defined as the longest time interval. If the slope was statistically significant (*P* < 0.05), stability duration was determined as the longest time interval at which the one-sided 95% CI of the predicted scores was within the 5.5 score threshold. The SD of the test group was required to remain below 5.5. Shelf-life was assigned to the latest time point within specification unless two consecutive failures occurred, in which case, the last compliant time point was used.

### Fresh-frozen equivalency

To determine the equivalency of TriVerity scores (bacterial, viral, and severity) from samples tested freshly or after freezing, we conducted a fresh-frozen equivalency study. After obtaining informed consent, 2.5 mL of whole blood was collected into PAXgene Blood RNA tubes and de-identified for testing at the study site. PAXgene tubes were tested at three conditions: (i) freshly upon collection at the site of patient enrollment, (ii) tested after 24 hours of storage at room temperature, and (iii) tested after freezing PAXgene Blood RNA tubes for 24 hours at −80°C. Test results were sent to Inflammatix for statistical analysis. The point estimate of the mean shift in bacterial, viral, and severity score was calculated for samples processed between time points T_0_ and T_1_ and T_0_ and T_2_. Equivalency was defined as a lack of significant and clinically meaningful differences in scores, i.e. *P*-value of <0.05 in conjunction with a shift of >5.5 score units (greater than 50% of interpretation band width) in TriVerity bacterial, viral, and illness severity scores. To calculate the *P*-value, a signed-rank Wilcoxon test was used (paired sample test between T_0_-T_1_ and T_0_-T_2_).

### Equivalency of TriVerity test results using NanoString nCounter or Myrna platforms

In addition to the analytical studies described, a method comparison study was performed to determine the intra- and interplatform concordance when processing PAXgene Blood RNA tubes on the NanoString nCounter platform (NanoString Technologies, Bothell, WA), which was used as a reference platform for classifier development and non-registrational clinical trials before the TriVerity test performed on the Myrna instrument became available. Whole blood was collected in PAXgene Blood RNA tubes, and samples were frozen following the draw, then thawed and processed on the nCounter instrument. Subsequently, after the TriVerity test and Myrna instrument were finalized, samples were tested. Variability factors included instruments, operators, cartridge lots, and days. We evaluated the reproducibility of the TriVerity scores compared to the reproducibility of the nCounter platform using the Pearson coefficient of correlation (PCC). Band concordance was calculated as the percentage of replicate samples assigned to the same or neighboring interpretation band.

### Operator survey

After the reproducibility and fresh-frozen equivalency studies and the SEPSIS-SHIELD registrational trial were completed, local operators were asked to complete an electronic survey on the ease of use of the TriVerity test and Myrna instrument.

### Statistical analysis

For reproducibility analysis, a nested model with site, instrument, and day was used, employing the REML criterion as the model objective and incorporating Grubbs’ outlier analysis. For fresh-frozen equivalency analyses, the Wilcoxon signed-rank test with confidence intervals of 95% was used to evaluate the significance of the shift in means. For equivalency studies, performance was determined using the PCC.

## RESULTS

### Reproducibility

Multi-site reproducibility performed using 90 replicate measurements per contrived sample demonstrated SDs below the acceptance criteria of <5.5 score units (Table 2) for all samples.

**TABLE 2:** Multi-site reproducibility results

Contrived sample^*a*^	TriVerity score	Mean score	Between day (SD)	Between instrument (SD)	Between site (SD)	Overall reproducibility (SD)
Sample A	Bacterial	38.17	0.00	1.25	1.82	4.91
	Viral	22.33	0.00	1.52	1.77	5.21
	Illness severity	37.48	1.35	1.63	0.00	3.65
Sample B	Bacterial	47.42	0.00	0.14	0.00	0.75
	Viral	6.24	0.59	0.35	0.00	1.91
	Illness severity	48.20	0.00	0.23	0.00	0.84
Sample C	Bacterial	1.13	0.05	0.16	0.07	0.51
	Viral	49.10	0.18	0.18	0.00	0.40
	Illness severity	6.86	0.00	0.65	0.00	1.56
Sample D	Bacterial	3.49	0.23	0.23	0.19	0.84
	Viral	3.17	0.00	0.44	0.00	1.01
	Illness severity	8.89	0.00	0.00	0.00	2.55

^*a*^ Expected results for pools A–D are described in Materials and Methods, “Reproducibility” section.

SD scores observed in the multi-site reproducibility study ranged from 0.00 to 1.35 between days, from 0.00 to 1.63 between instruments, and from 0.00 to 1.82 between sites for all contrived sample pools (bacterial, viral, and illness severity) meeting the preset acceptance criteria. Overall reproducibility SD ranged from 0.51 to 2.55 for contrived samples B, C, and D and between 4.91 and 5.21 for contrived sample A, meeting acceptance criteria.

### Precision

The overall SD for bacterial, viral, and illness severity scores was 2.71, 2.89, and 2.93, respectively (Table 3) and met acceptance criteria for each band for each score (SD <5.5 score units).

**TABLE 3:** Precision

Band	Imprecision SD			Number of samples per band		
	Bacterial	Viral	Severity	Bacterial	Viral	Severity
Very low	1.59	1.44	1.72	24	33	35
Low	3.21	4.32	3.89	39	22	25
Moderate	2.9	3.56	3.61	25	31	30
High	3.69	3.58	2.92	22	20	33
Very high	1.22	0.85	1.38	28	32	15
Overall	2.71	2.89	2.93	138	138	138

### Lot-to-lot reproducibility

Lot-to-lot reproducibility, as assessed based on 24 cartridges per lot tested in 6 replicates per each of the 4 different sample types, revealed SDs below the acceptance criteria of <5.5 (Table 4). There was complete agreement (100%) for all samples and scores, except for the bacterial score in sample A. The percent agreement for the bacterial score in sample A could be either 66% or 100%, based on how we decided to resolve ties in the counts of six replicates from one of the lots. Overall, the Fleiss kappa for the 12 score and sample combinations across the 3 lots was 0.915 when the tie breaker was treated as distinct from the modal score.

**TABLE 4:** Lot-to-lot reproducibility

Contrived sample	TriVerity score	Mean score	Reproducibility between lots (SD)	Overall SD
Sample A	Bacterial	33.56	0.0	4.7
	Viral	27.33	0.0	3.5
	Severity	37.5	1.1	2.7
Sample B	Bacterial	47.61	0.0	0.8
	Viral	6.28	0.0	1.8
	Severity	47.94	0.0	0.6
Sample C	Bacterial	1.00	0.0	0.0
	Viral	49.17	0.0	0.4
	Severity	7.33	0.4	1.2
Sample D	Bacterial	3.33	0.0	0.5
	Viral	3.94	0.0	0.7
	Severity	9.33	1.0	2.5

### Analytical sensitivity

The lowest concentration at which 100% (20/20) of the measured replicates tested demonstrated measurable amplification for the 29 informative genes and 3 housekeeping genes was 1 × 10^6^ cp/mL (Table S5). At a concentration of 5 × 10^5^ cp/mL, 31/32 genes showed 100% amplification. Amplification ranged from 95% to 100% for 25 genes at a concentration of 1 × 10^5^ cp/mL; 5 genes (HLA-DMB, IFI27, IFI44L, S100A12, and TDRD9) amplified in 90% of replicates, whereas XAF1 and IL1R2 amplified in 80% and 40% of replicates, respectively.

### Linearity

Linearity testing was conducted across the concentration range of 1 × 10⁶–1 × 10⁹ copies/mL for all target genes (Table S6). The regression analysis results showed that all genes displayed strong linearity, as indicated by Pearson correlation coefficients ranging from 0.943 to 0.997. The slopes for each gene varied from −1.57 to −2.95, and intercepts ranged from 27.59 to 43.84. Importantly, all genes passed the allowable deviation from linearity criterion of 5%, confirming the reliability of the linear model across the tested concentration range (Table S6). These results demonstrate that the system is linear for all the 32 genes within the range of 1 × 10^6^–1 × 10^9^ copies/mL, considering an ADL of 5%.

### Limit of detection and quantitation

All leuko-reduced blood (blanks) runs did not provide a result. Both the LoQ and the limit of detection were determined to be equivalent to 500 cells/µL (rounded up from 499 and 432 cells/µL) for both sample pools (Table 5). For pool A, replicates of diluted samples of 499 cells/µL provided results in 100% of tests, replicates of diluted samples of 374 cells/µL provided results in 90% of tests, replicates of diluted samples of 249 cells/µL provided results in 89% of tests, and replicates of diluted samples of 125 cells/µL provided results in 60% of tests. For pool B, replicates of diluted samples of 432 cells/µL provided results in 100% of tests, replicates of diluted samples of 324 cells/µL provided results in 100% of tests, replicates of diluted samples of 216 cells/µL provided results in 95% of tests, and replicates of diluted samples of 108 cells/µL provided results in 65% of tests.

**TABLE 5:** Limits of quantification for blood pools

Sample type	Cells/µL	*N*	Average score (SD)			
			Bacterial	Viral	Illness severity	Amplification
Leuko-reduced blood	N/A	10	N/A	N/A	N/A^*a*^	0%
Viral pool	1,995	10	15.30 (2.8)	42.30 (1.3)	12.80 (3.8)	100%
	499	20	16.10 (4.1)	38.50 (4.4)	11.29 (3.3)	100%
	374	20	13.89 (2.8)	38.50 (3.7)	11.50 (4.9)	90%
	249	20	12.82 (3.3)	36.59 (5.1)	10.41 (5.8)	89%
	125	20	13.08 (3.0)	29.08 (7.4)	10.58 (4.3)	60%
Bacterial/severity pool	1,296	10	45.70 (1.2)	7.20 (1.6)	42.00 (3.4)	100%
	432	20	44.40 (1.4)	8.35 (1.9)	39.80 (2.7)	100%
	324	20	44.19 (1.0)	8.95 (1.8)	40.24 (3.0)	100%
	216	20	42.53 (2.3)	11.37 (3.3)	36.42 (4.7)	95%
	108	20	38.85 (4.6)	13.54 (5.5)	30.38 (6.6)	65%

^*a*^ N/A, not applicable.

### Interference and cross-reactivity

Results of replicate testing of each potential interferent commonly encountered in whole blood specimens compared to original contrived sample controls are presented in Table S7. No interference was observed for any of the 17 potential interferents tested.

No cross-reactivity with gDNA spiked at 10 ng/µL was observed. Absolute scores for the control samples and gDNA-spiked samples showed differences of 0.83 and 0.33 for the bacterial score, 0.5 and 0.66 for the viral score, and 1.83 and 0.33 for the illness severity score (Table S8). All TriVerity results obtained in control and gDNA-spiked samples fell into the same TriVerity interpretation band except for one viral score and one bacterial score, both scores crossing cutoffs by only one interpretation band. No amplification signal was observed for samples spiked with 20 ng/µL human gDNA template.

### TriVerity cartridge stability

Cartridges stored for 3, 6, and 9 months at 15°C and 30°C, as well as for 3, 6, 9, and 12 months at an accelerated temperature of 37°C, demonstrated no significant degradation in performance for both sample panel members tested. Stability criteria were met across all conditions, with SD and amplitude changes remaining below the predefined acceptance threshold of 5.50 score units (Table S9). *P*-values exceeded 0.05 for all tested conditions (temperatures and samples), except for contrived sample C at 30°C, where a *P*-value of 0.0119 was observed. Despite this, the regression slope was zero, and extrapolation to 18 months (study duration) indicated that the amplitude of change remained within the acceptance criteria. These findings confirm the stability of the cartridges under the tested storage conditions.

### Fresh-frozen equivalency

**Fig 2:**
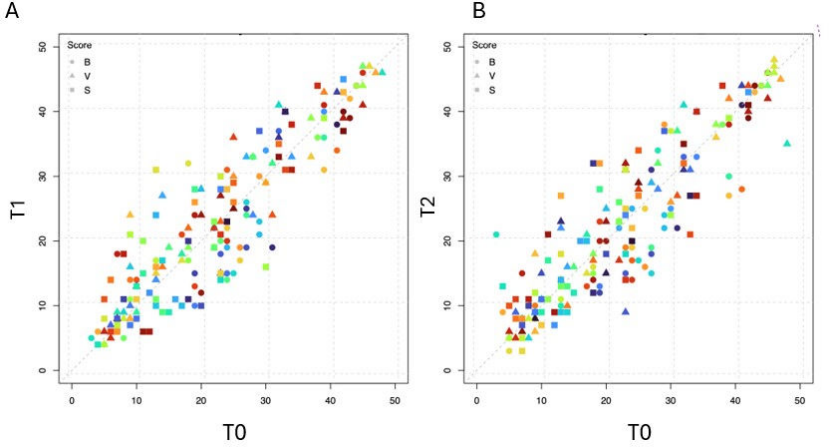
Scatter plot of TriVerity bacterial, viral, and illness severity scores obtained at different time points and temperature conditions. (**A**) Blood collection (T0) vs 24 hours at room temperature (T1); (**B**) blood collection (T0) vs 24 hours at −80°C (T2). Bacterial scores are indicated by circles, viral scores are indicated by triangles, and illness severity scores are indicated by squares and colored for individual patients.

The TriVerity bacterial, viral, and illness severity scores obtained at time points T_0_ and T_1_ and between time points T_0_ and T_2_ were compared. There was a shift in TriVerity bacterial scores between the time of collection (T_0_) and after 24 hours at room temperature (T_1_) (*P* < 0.02) and between T_0_ and after 24 hours at −80°C (T_2_) (*P* < 0.002). However, the shift was lower than 5.5 score units, therefore meeting the predefined acceptance criteria for fresh-frozen equivalency. The shift in TriVerity viral scores (*P* = 0.005) for comparing T_0_ vs T_1_ was below 5.5 score units, also meeting the predefined acceptance criteria. We did not observe significant differences in the Viral scores between T_0_ and T_2_. Lastly, the shift in illness severity scores was not statistically significant for the comparison of T_0_ vs T_1_ (*P* = 0.21) and T_0_ vs T_2_ (*P* = 0.13). Relative to T_0_, in both T_1_ and T_2_, only two bacterial, one viral, and two severity scores shifted to non-adjacent bands (Fig. S1). TriVerity scores obtained between T_0_ and T_1_ are depicted in a scatter plot in Fig. 2A, whereas the scores obtained between T_0_ and T_2_ are depicted in Fig. 2B.

### Samples with low cellularity/low RNA

To amplify host genes, the TriVerity test uses RNA extracted from whole blood collected in PAXgene Blood RNA tubes. The test generates a code (“insufficient RNA”) if blood samples have insufficient cellularity/insufficient RNA to amplify the target genes. During the SEPSIS-SHIELD registrational study performed in ED patients with suspected acute infection or suspected sepsis, this code was observed in 14 of 1,253 (1.1%) runs performed. While the overall rates of samples with insufficient cellularity/insufficient RNA were low, the code represents a beneficial feature by invalidating samples quickly but also potentially being of medical interest to the clinician.

### Method comparison TriVerity test on Myrna vs NanoString nCounter

**Fig 3:**
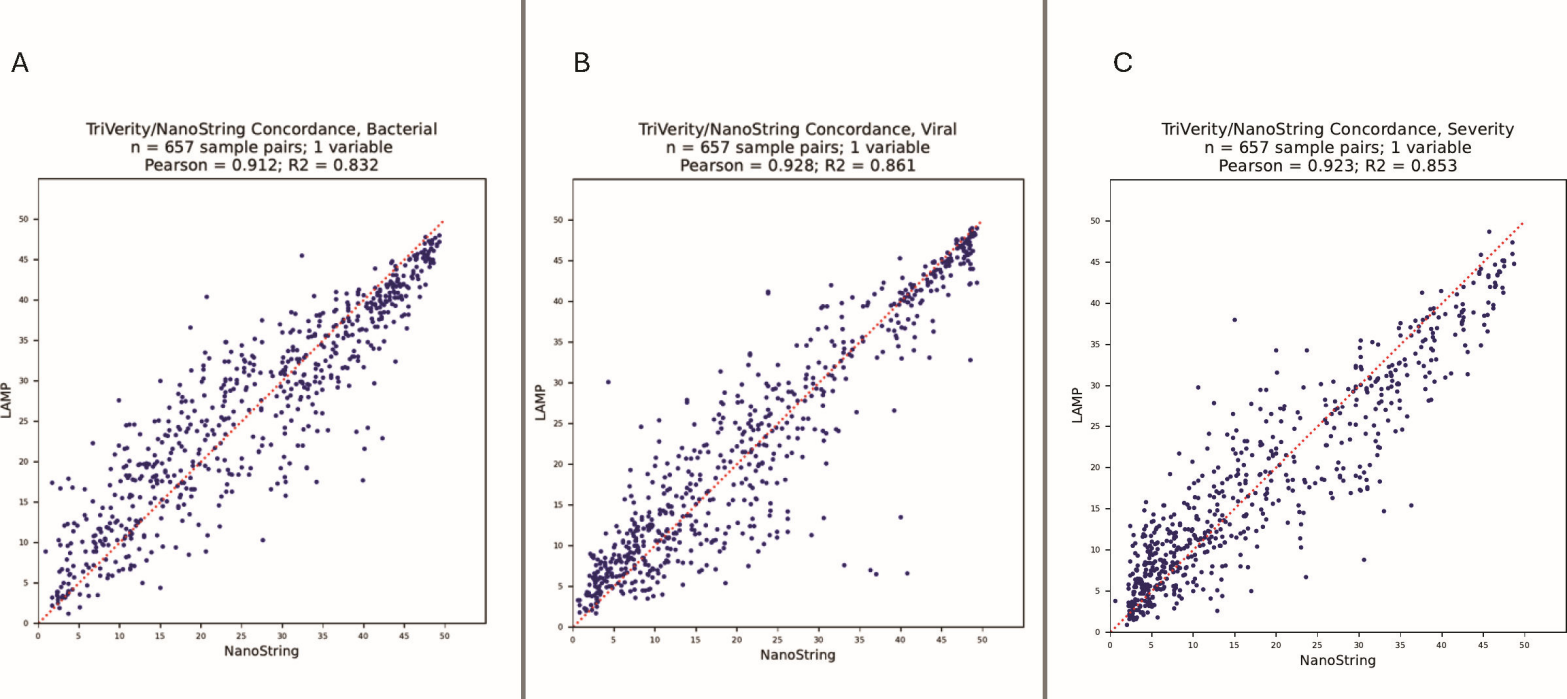
Concordance of TriVerity bacterial (**A**), viral (**B**), and illness severity (**C**) scores obtained on the Myrna (LAMP) vs NanoString nCounter platform.

Scores obtained with the TriVerity test on the Myrna instrument were compared to scores generated from processing the same samples on the NanoString nCounter^®^ reference platform (Fig. 3; Table S11). For 657 paired PAXgene samples processed on both platforms, the Myrna-to-NanoString PCC was high, 0.91 for bacterial scores, 0.93 for viral scores, and 0.92 for severity scores. The percentage of TriVerity replicates assigned to the same or neighboring interpretation band on the Myrna and NanoString nCounter platform was 96% for bacterial scores, 97% for viral scores, and 96% for severity scores. These results indicate that results obtained using the same or earlier classifier versions on the NanoString nCounter platform are equivalent to those generated on the commercially available TriVerity test performed on the Myrna platform.

### Operator survey

**Fig 4:**
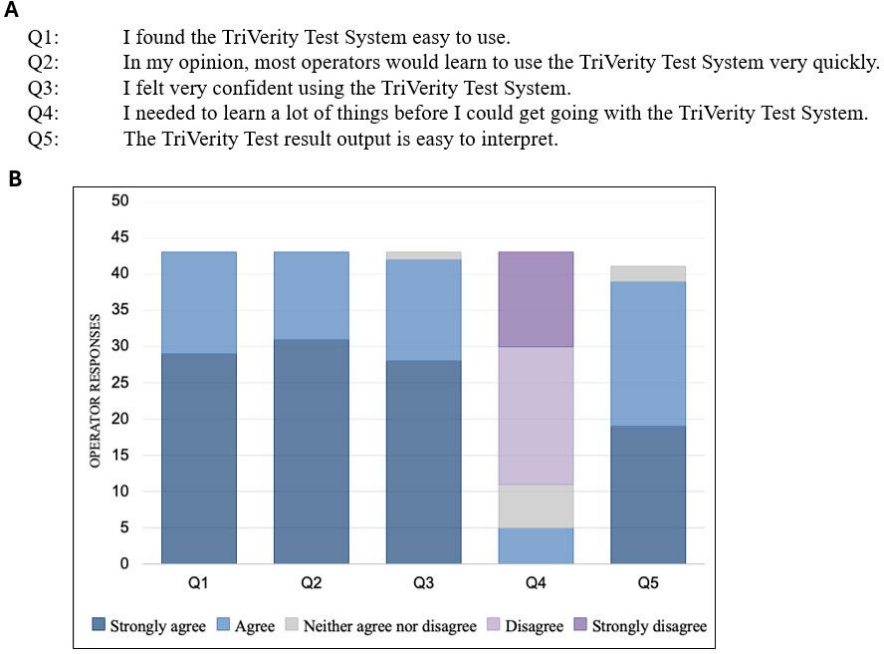
Operator survey responses about using the TriVerity test system. Operators were surveyed on the test’s ease of use and instrument upon completion of studies: (**A**) selected survey questions and (**B**) survey responses (*n* = 43).

In the present analytical studies and registrational trial, 43 operators participated and completed the electronic survey about their experience with the TriVerity test and Myrna instrument (Fig. 4A and B). The majority of operators performed between 6 and 20 TriVerity tests. Upon completion of the studies, operators reported an overall positive experience: they found the instructions for use easy to learn and were confident running the test. All operators agreed or strongly agreed that the TriVerity test system was easy to use.

## DISCUSSION

Implementation of clinical diagnostics requires not only the demonstration of clinical accuracy in non-registrational (13–16) and registrational trials (23) but also rigorous analytical validation. The analytical studies described here demonstrate that the TriVerity test system meets or exceeds analytical validation metrics, including reproducibility, precision, analytical sensitivity, interference, cross-reactivity, fresh-frozen equivalency, stability, and failure rate.

Across multiple testing sites, the TriVerity test system demonstrated high reproducibility. The precision analysis showed overall SDs for bacterial, viral, and severity scores of 2.71, 2.89, and 2.93, respectively, each falling within the predefined acceptable limits. These results indicate that the TriVerity test system maintains consistent performance despite variability in testing conditions, including site, operator, instrument, and cartridge lot. Analytical sensitivity studies confirmed that 100% of replicates amplified the 29 informative genes and 3 housekeeping genes at concentrations as low as 10^6^ cp/mL, and the majority (18/32 genes) amplified at 10^5^ cp/mL, highlighting the test’s broad dynamic range and detection capability. There was no interference observed for 17 interferents relevant to the intended use population, including a broad range of antibiotics, biomarkers, and serum proteins chosen to reflect potential interferents found in the blood of patients in the intended use population (suspected acute infection or suspected sepsis in the emergency department). Interference with genomic DNA was ruled out, supporting the specificity of the test’s RNA amplification. Our analytical validation results are in line with findings reported for studies of other multiplex mRNA host response tests (24, 25).

A benefit of PAXgene RNA tubes is that they stabilize intracellular RNA immediately upon blood collection, enabling reliable long-term storage and preservation of gene expression profiles (26). This capability enabled the collection of samples for classifier (IMX-BVN-4/SEV-4) development, clinical validation, cartridge and instrument development, and the analytical studies conducted here. Sample stability and equivalency studies demonstrated consistency of the TriVerity test results when whole blood collected in PAXgene Blood RNA tubes was tested fresh, after 24 hours at room temperature, or following storage at −80°C. More than 96% of the TriVerity scores remained in the original or adjacent interpretation band. Since freeze-thaw cycles are known to affect clinical samples, these findings support frozen storage for repeat or delayed testing of samples after storage in clinical or research settings, as indicated in the instructions for use for the PAXgene Blood RNA tube (27). The cartridge stability study revealed real-time shelf-life for 9 months at room temperature, with ongoing studies supporting up to 12 months. We further demonstrated that samples processed on the Myrna instrument yield results equivalent to those of the NanoString nCounter. This supports the transferability of data across assay platforms, as many clinical study samples were previously processed using the NanoString nCounter platform, a reference platform for gene expression studies, during development of the TriVerity test system (13–16, 28, 29).

In addition to analytical performance, the operational aspects of new tests are essential considerations for supporting their integration into routine clinical and laboratory workflows. The ease of collection of blood into the PAXgene RNA vacutainer, RNA stabilization at different temperatures, and the elimination of additional processing steps, such as centrifugation at the collection location and storage on ice prior to RNA purification, facilitate standardization of the workflow from collection to test performance, while reducing variability that can be introduced by different operators (27). In addition, the operator survey revealed an overall positive operator experience with the TriVerity test and Myrna instrument, i.e., overall ease of use and the ease of interpretation of the TriVerity result outputs.

Results presented here provide further performance details for the TriVerity test in addition to the information provided in the package insert, thus supporting the development of verification plans for laboratories (30). As TriVerity results are semi-quantitative scores that fall into interpretation bands rather than the dichotomous results typical of molecular infectious disease tests (detected/not detected or positive/negative), verification may be complex for laboratories due to the scarcity of remnant samples and/or simple reference standards. We thus strongly advocate for the use of contrived samples during validation and verification at the end-user’s laboratory.

There are limitations worth highlighting. First, although many of the commonly encountered interferents were tested, all clinically relevant conditions (e.g., lipemia, hyperproteinemia, etc.) affecting the sample or collection were not included. Second, operator usability was qualitatively assessed without standardized metrics or response rates, which limits the strength of the conclusions drawn.

In conclusion, the TriVerity system demonstrates analytical performance characteristics that meet or exceed regulatory and laboratory standards. Along with its ease of use, TriVerity could prove to be a valuable tool for supporting clinical care decisions in acute care settings.

## SUPPLEMENTAL MATERIAL

Supplemental figures and tables jcm.00352-25-s0001.docx Fig. S1, and Tables S1 to S11.

ASM does not own the copyrights to Supplemental Material that may be linked to, or accessed through, an article. The authors have granted ASM a non-exclusive, world-wide license to publish the Supplemental Material files. Please contact the corresponding author directly for reuse.

## References

[1] Mi MY, Klompas M, Evans L. 2019. Early administration of antibiotics for suspected sepsis. N Engl J Med 380:593–596. doi:10.1056/NEJMclde1809210 30726686

[2] Singer M, Deutschman CS, Seymour CW, Shankar-Hari M, Annane D, Bauer M, Bellomo R, Bernard GR, Chiche J-D, Coopersmith CM, Hotchkiss RS, Levy MM, Marshall JC, Martin GS, Opal SM, Rubenfeld GD, van der Poll T, Vincent J-L, Angus DC. 2016. The third international consensus definitions for sepsis and septic shock (sepsis-3). JAMA 315:801–810. doi:10.1001/jama.2016.0287 26903338 PMC4968574

[3] Evans L, Rhodes A, Alhazzani W, Antonelli M, Coopersmith CM, French C, Machado FR, Mcintyre L, Ostermann M, Prescott HC. 2021. Surviving sepsis campaign: international guidelines for management of sepsis and septic shock 2021. Crit Care Med 49:1063–1143. doi:10.1097/CCM.0000000000005337 34605781

[4] Gaieski DF, Mikkelsen ME, Band RA, Pines JM, Massone R, Furia FF, Shofer FS, Goyal M. 2010. Impact of time to antibiotics on survival in patients with severe sepsis or septic shock in whom early goal-directed therapy was initiated in the emergency department. Crit Care Med 38:1045–1053. doi:10.1097/CCM.0b013e3181cc4824 20048677

[5] Ferrer R, Martin-Loeches I, Phillips G, Osborn TM, Townsend S, Dellinger RP, Artigas A, Schorr C, Levy MM. 2014. Empiric antibiotic treatment reduces mortality in severe sepsis and septic shock from the first hour: results from a guideline-based performance improvement program. Crit Care Med 42:1749–1755. doi:10.1097/CCM.0000000000000330 24717459

[6] Rhee C, Chiotos K, Cosgrove SE, Heil EL, Kadri SS, Kalil AC, Gilbert DN, Masur H, Septimus EJ, Sweeney DA, Strich JR, Winslow DL, Klompas M. 2021. Infectious diseases society of America position paper: recommended revisions to the national severe sepsis and septic shock early management bundle (SEP-1) sepsis quality measure. Clin Infect Dis 72:541–552. doi:10.1093/cid/ciaa059 32374861 PMC8189682

[7] Prescott HC, Iwashyna TJ. 2019. Improving sepsis treatment by embracing diagnostic uncertainty. Ann Am Thorac Soc 16:426–429. doi:10.1513/AnnalsATS.201809-646PS 30883190 PMC6441693

[8] Ducharme J, Self WH, Osborn TM, Ledeboer NA, Romanowsky J, Sweeney TE, Liesenfeld O, Rothman RE. 2020. A multi-mRNA host-response molecular blood test for the diagnosis and prognosis of acute infections and sepsis: proceedings from a clinical advisory panel. J Pers Med 10:266. doi:10.3390/jpm10040266 33297498 PMC7762405

[9] Gunsolus IL, Sweeney TE, Liesenfeld O, Ledeboer NA. 2019. Diagnosing and managing sepsis by probing the host response to infection: advances, opportunities, and challenges. J Clin Microbiol 57:e00425-19. doi:10.1128/JCM.00425-19 31043466 PMC6595443

[10] Jain S, Self WH, Wunderink RG, CDC EPIC Study Team. 2015. Community-acquired pneumonia requiring hospitalization. N Engl J Med 373:2382. doi:10.1056/NEJMc1511751 26650159 PMC9338768

[11] Ohnuma T, Chihara S, Costin B, Treggiari MM, Bartz RR, Raghunathan K, Krishnamoorthy V. 2023. Association of appropriate empirical antimicrobial therapy with in-hospital mortality in patients with bloodstream infections in the US. JAMA Netw Open 6:e2249353. doi:10.1001/jamanetworkopen.2022.49353 36598788 PMC9857618

[12] Liu Z, Meng Z, Li Y, Zhao J, Wu S, Gou S, Wu H. 2019. Prognostic accuracy of the serum lactate level, the SOFA score and the qSOFA score for mortality among adults with Sepsis. Scand J Trauma Resusc Emerg Med 27:51. doi:10.1186/s13049-019-0609-3 31039813 PMC6492372

[13] Galtung N, Diehl-Wiesenecker E, Lehmann D, Markmann N, Bergström WH, Wacker J, Liesenfeld O, Mayhew M, Buturovic L, Luethy R, Sweeney TE, Tauber R, Kappert K, Somasundaram R, Bauer W. 2022. Prospective validation of a transcriptomic severity classifier among patients with suspected acute infection and sepsis in the emergency department. Eur J Emerg Med 29:357–365. doi:10.1097/MEJ.0000000000000931 35467566 PMC9432813

[14] Bauer W, Kappert K, Galtung N, Lehmann D, Wacker J, Cheng HK, Liesenfeld O, Buturovic L, Luethy R, Sweeney TE, Tauber R, Somasundaram R. 2021. A novel 29-messenger RNA host-response assay from whole blood accurately identifies bacterial and viral infections in patients presenting to the emergency department with suspected infections: a prospective observational study. Crit Care Med 49:1664–1673. doi:10.1097/CCM.0000000000005119 34166284 PMC8439671

[15] Safarika A, Wacker JW, Katsaros K, Solomonidi N, Giannikopoulos G, Kotsaki A, Koutelidakis IM, Coyle SM, Cheng HK, Liesenfeld O, Sweeney TE, Giamarellos-Bourboulis EJ. 2021. A 29-mRNA host response test from blood accurately distinguishes bacterial and viral infections among emergency department patients. Intensive Care Med Exp 9:31. doi:10.1186/s40635-021-00394-8 34142256 PMC8211458

[16] Kostaki A, Wacker JW, Safarika A, Solomonidi N, Katsaros K, Giannikopoulos G, Koutelidakis IM, Hogan CA, Uhle F, Liesenfeld O, Sweeney TE, Giamarellos-Bourboulis EJ. 2022. A 29-mRNA host response whole-blood signature improves prediction of 28-day mortality and 7-day intensive care unit care in adults presenting to the emergency department with suspected acute infection and/or sepsis. Shock 58:224–230. doi:10.1097/SHK.0000000000001970 36125356 PMC9512237

[17] Liesenfeld O, Arora S, Aufderheide T, Clements C, DeVos E, Fischer M, Giamarellos-Bourboulis E, House S, Humphries R, Gill JK, et al.. 2024. Rapid and accurate diagnosis and prognosis of acute infections and sepsis from whole blood using host response mRNA amplification and result interpretation by machine-learning classifiers. Res Square. doi:10.21203/rs.3.rs-5194992/v1

[18] Lu J, Ter Voert MA, Ünal M, Whitfield NN, Liesenfeld O, Ter Maaten JC, Sweeney TE, Bouma HR. 2025. Early sepsis recognition: a pilot study using a rapid high-multiplex host-response mRNA diagnostic test. Intensive Care Med Exp 13:21. doi:10.1186/s40635-025-00735-x 39982589 PMC11845329

[19] Buturovic L, Mayhew M, Luethy R, Choi K, Midic U, Damaraju N, Hasin-Brumshtein Y, Pratap A, Adams RM, Fonseca J, Srinath A, Fleming P, Pereira C, Liesenfeld O, Khatri P, Sweeney TE. 2024. Development of machine learning classifiers for blood-based diagnosis and prognosis of suspected acute infections and sepsis. ArXiv. doi:10.48550/arXiv.2407.02737

[20] He YD, Wohlford EM, Uhle F, Buturovic L, Liesenfeld O, Sweeney TE. 2021. The optimization and biological significance of a 29-host-immune-mRNA panel for the diagnosis of acute infections and sepsis. J Pers Med 11:735. doi:10.3390/jpm11080735 34442377 PMC8402342

[21] McEnroe RJ. 2014. CLSI EP05: evaluation of precision of quantitative measurement procedures

[22] Pierson-Perry JF. 2012. CLSI EP17: evaluation of detection capability for clinical laboratory measurement procedures

[23] FDA Inc. 2025. 510(k) Premarket notification, TriVerity test

[24] FDA. 2017. 510(k) Substantial equivalence determination decision summary SeptiCyte lab

[25] FDA. 2021. 510(k) Substantial equivalence determination decision summary SeptiCyte raapid

[26] Tang R, She Q, Lu Y, Yin R, Zhu P, Zhu L, Zhou M, Zheng C. 2019. Quality control of RNA extracted from PAXgene blood RNA tubes after different storage periods. Biopreserv Biobank 17:477–482. doi:10.1089/bio.2019.0029 31343263

[27] Qiagen. PAXgene blood RNA kit (IVD) for RNA isolation

[28] Brakenridge SC, Starostik P, Ghita G, Midic U, Darden D, Fenner B, Wacker J, Efron PA, Liesenfeld O, Sweeney TE, Moldawer LL. 2021. A transcriptomic severity metric that predicts clinical outcomes in critically ill surgical sepsis patients. Crit Care Explor 3:e0554. doi:10.1097/CCE.0000000000000554 34671746 PMC8522866

[29] Brakenridge SC, Chen U-I, Loftus T, Ungaro R, Dirain M, Kerr A, Zhong L, Bacher R, Starostik P, Ghita G, Midic U, Darden D, Fenner B, Wacker J, Efron PA, Liesenfeld O, Sweeney TE, Moldawer LL. 2022. Evaluation of a multivalent transcriptomic metric for diagnosing surgical sepsis and estimating mortality among critically ill patients. JAMA Netw Open 5:e2221520. doi:10.1001/jamanetworkopen.2022.21520 35819783 PMC9277492

[30] Yusuf E, Schijffelen MJ, Leeflang M. 2024. How to verify and validate a clinical microbiology test before it can be used in routine diagnostics: a practical guide. Clin Microbiol Infect 30:1261–1269. doi:10.1016/j.cmi.2024.06.028 38977077

