# Supplementary material for: Analytical evaluation of TriVerity, a rapid diagnostic and prognostic host gene expression test performed on the Myrna instrument using RT-LAMP
Source: J Clin Microbiol. 2025 Aug 27;63(10):e00352-25. doi: 10.1128/jcm.00352-25 (PMC12506079; doi:10.1128/jcm.00352-25)
Supplement: Supplemental figures and tables — Fig. S1, and Tables S1 to S11. [file jcm.00352-25-s0001.docx]

**Analytical evaluation of TriVerity, a rapid diagnostic and prognostic host gene expression test performed on the Myrna instrument using RT-LAMP**

**AUTHORS:** Claudia Figueiredo-Pereira¹†, Paul Fleming¹†, Mikaela Nicole Alganes¹, Ran Bi¹, Ana Mafalda Cavaleiro¹, Diogo Cruz¹, Carlota Cunha-Matos¹, Margarita Davalos-Arias¹, Dana Farkas¹, Yehudit Hasin¹, Kevin Hu¹, Christos Kampouridis¹, Ragheb El Khaja¹, Graciano Leal¹, Jingyi Lu^3^, Rita Madeira¹, Michael Mayhew¹, Breana McBryde^2^, Daniela C. Oliveira¹, Anna Passernig¹, Davion Pendleton¹, Elizabeth Popp¹, Shailee Rasania¹, Cristina Rebelo¹, Ana Santiago¹, Joshua R. Shak¹, Vera P. Silva¹, Ambika Srinath¹, Timothy E. Sweeney¹, Rodrigo Vieira¹, Thang Vu¹, Chris Wilson¹, Boris Zybin¹, Natalie N. Whitfield¹#, Oliver Liesenfeld¹, Hjalmar R. Bouma^3,4,5^*, Richard E. Rothman^2^*, Edward A. Michelson^6*^, Joao Fonseca^1*^

Table of Contents

[Supplementary Figures 2](#_Toc203236612)

[Supplementary Figure 1. 2](#_Toc203236613)

[Supplementary Tables 4](#_Toc203236614)

[Supplementary Table 1. Instrument and cartridge parameters to determine lot-to-lot reproducibility 4](#_Toc203236615)

[Supplementary Table 2. Sample volumes and white blood cell counts in blood pools used for limit of quantitation testing 5](#_Toc203236616)

[Supplementary Table 3. Endogenous and exogenous interfering substances preparation 6](#_Toc203236617)

[Supplementary Table 4. Analytical sensitivity and linearity sample preparation 7](#_Toc203236618)

[Supplementary Table 5. Analytical sensitivity 8](#_Toc203236619)

[Supplementary Table 6: Linearity 9](#_Toc203236620)

[Supplementary Table 7: Endogenous and exogenous interferences 10](#_Toc203236621)

[Supplementary Table 8. Cross reactivity with genomic DNA 11](#_Toc203236622)

[Supplementary Table 9. TriVerity Cartridge stability 12](#_Toc203236623)

[Supplementary Table 10. TriVerity Fresh-Frozen Equivalency Demonstrated using Score Mean Shift 13](#_Toc203236624)

[Supplementary Table 11: Intra-and inter-platform concordance of the Myrna Instrument and NanoString nCounter for TriVerity Bacterial and Viral scores. 13](#_Toc203236625)

# Supplementary Figures

**Supplementary Figure 1.** Scatter plot of TriVerity Bacterial, Viral, and Illness Severity scores obtained at different time points and temperature conditions. Top panels: Bacterial scores; Middle panels: Viral scores; Bottom panels: Illness Severity scores. Left panels are T_0_ vs. 24 hrs at room temperature (T_1_) and Right panels are T_0_ vs. 24 hrs at -80 °C (T_2_). Middle panels: Viral scores for T_0_ vs. 24 hrs at room temperature (T_1_), left and T_0_ vs. 24 hrs at -80 °C (T_2_), right. Each point represents two scores for an individual patient sample.


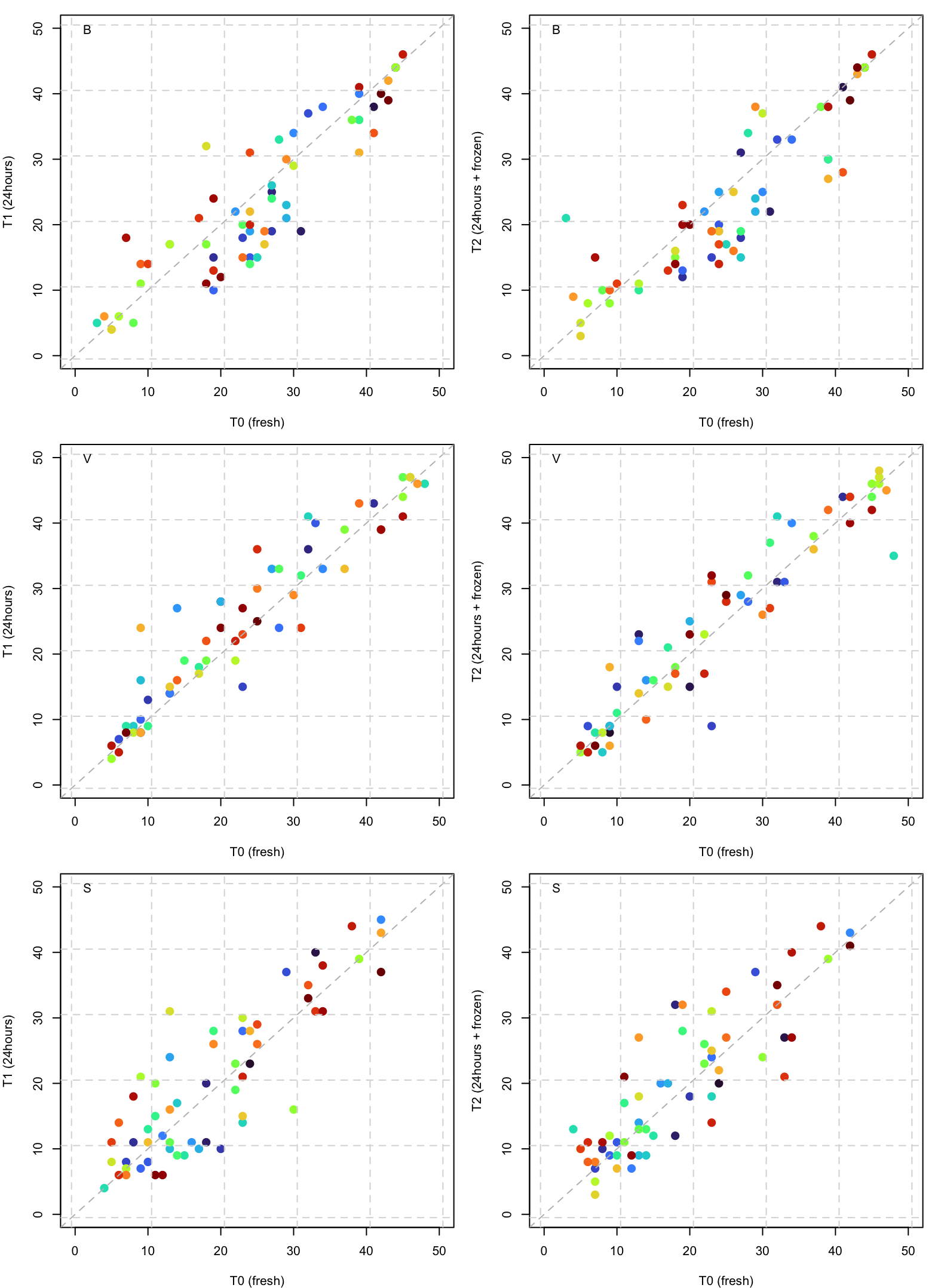


# Supplementary Tables

## Supplementary Table 1. Instrument and cartridge parameters to determine lot-to-lot reproducibility

| **Testing Round** | **Instrument 1** | **Instrument 2** | **Instrument 3** | **Instrument 4** |
| --- | --- | --- | --- | --- |
| 1 | Sample A  Cartridge Lot A | Sample B  Cartridge Lot A | Sample C  Cartridge Lot A | Sample D  Cartridge Lot A |
| 2 | Sample A  Cartridge Lot B | Sample B  Cartridge Lot B | Sample C  Cartridge Lot B | Sample D  Cartridge Lot B |
| 3 | Sample A  Cartridge Lot C | Sample B  Cartridge Lot C | Sample C  Cartridge Lot C | Sample D  Cartridge Lot C |
| 4 | Sample A  Cartridge Lot A | Sample B  Cartridge Lot A | Sample C  Cartridge Lot A | Sample D  Cartridge Lot A |
| 5 | Sample A  Cartridge Lot B | Sample B  Cartridge Lot B | Sample C  Cartridge Lot B | Sample D  Cartridge Lot B |
| 6 | Sample A  Cartridge Lot C | Sample B  Cartridge Lot C | Sample C  Cartridge Lot C | Sample D  Cartridge Lot C |
| 7 | Sample A  Cartridge Lot A | Sample B  Cartridge Lot A | Sample C  Cartridge Lot A | Sample D  Cartridge Lot A |
| 8 | Sample A  Cartridge Lot B | Sample B  Cartridge Lot B | Sample C  Cartridge Lot B | Sample D  Cartridge Lot B |
| 9 | Sample A  Cartridge Lot C | Sample B  Cartridge Lot C | Sample C  Cartridge Lot C | Sample D  Cartridge Lot C |
| 10 | Sample A  Cartridge Lot A | Sample B  Cartridge Lot A | Sample C  Cartridge Lot A | Sample D  Cartridge Lot A |
| 11 | Sample A  Cartridge Lot B | Sample B  Cartridge Lot B | Sample C  Cartridge Lot B | Sample D  Cartridge Lot B |
| 12 | Sample A  Cartridge Lot C | Sample B  Cartridge Lot C | Sample C  Cartridge Lot C | Sample D  Cartridge Lot C |
| 13 | Sample A  Cartridge Lot A | Sample B  Cartridge Lot A | Sample C  Cartridge Lot A | Sample D  Cartridge Lot A |
| 14 | Sample A  Cartridge Lot B | Sample B  Cartridge Lot B | Sample C  Cartridge Lot B | Sample D  Cartridge Lot B |
| 15 | Sample A  Cartridge Lot C | Sample B  Cartridge Lot C | Sample C  Cartridge Lot C | Sample D  Cartridge Lot C |
| 16 | Sample A  Cartridge Lot A | Sample B  Cartridge Lot A | Sample C  Cartridge Lot A | Sample D  Cartridge Lot A |
| 17 | Sample A  Cartridge Lot B | Sample B  Cartridge Lot B | Sample C  Cartridge Lot B | Sample D  Cartridge Lot B |
| 18 | Sample A  Cartridge Lot C | Sample B  Cartridge Lot C | Sample C Cartridge Lot C | Sample D  Cartridge Lot C |

## Supplementary Table 2. Sample volumes and white blood cell counts in blood pools used for limit of quantitation testing

| **Sample** | **WBC Counts (cells/ µL)** | **Volume of Clinical Pool**  **(mL)** | | | **Previous Concentration (mL)** | **Leukocyte- Reduced Blood (mL)** | | **Total Volume (mL)** | | | **Total Runs**  **(N)** |  |
| --- | --- | --- | --- | --- | --- | --- | --- | --- | --- | --- | --- | --- |
| Pool Alone | N/A | | N/A | N/A | | | 20 | | 20 | 10 | | |
| Pool A | 1995 | | 30.00 | N/A | | | N/A | | 30.00 | 10 | | |
|  | 748 | | 18.38 | N/A | | | 30.62 | | 49.00 | 10 | | |
|  | 499 | | N/A | 30.67 | | | 15.33 | | 46.00 | 20 | | |
|  | 374 | | N/A | 27.76 | | | 9.24 | | 37.00 | 20 | | |
|  | 249 | | N/A | 18.67 | | | 9.33 | | 28.00 | 20 | | |
|  | 125 | | N/A | 10.00 | | | 10.00 | | 20.00 | 20 | | |
| Pool B | 1296 | | 30.00 | N/A | | | N/A | | 30.00 | 10 | | |
|  | 648 | | 20.00 | N/A | | | 20.00 | | 40.00 | 10 | | |
|  | 432 | | N/A | 30.00 | | | 15.00 | | 45.00 | 20 | | |
|  | 324 | | N/A | 27.00 | | | 9.00 | | 36.00 | 20 | | |
|  | 216 | | N/A | 18.67 | | | 9.33 | | 28.00 | 20 | | |
|  | 108 | | N/A | 10.00 | | | 10.00 | | 20.00 | 20 | | |

## Supplementary Table 3. Endogenous and exogenous interfering substances preparation

| **Substances** | **Concentration in PAXgene blood RNA tube (pre-RNA extraction)** | **Equivalent concentration in whole blood (*)** |
| --- | --- | --- |
| Bilirubin | 400 mg/L | 1504 mg/L |
| Hemoglobin | 10 g/L | 37.6 g/L |
| Rheumatoid Factor | 45 U/mL | 169.2 U/mL |
| Triglycerides | 2000 mg/dL | 7520 mg/dL |
| Albumin | 5 g/dL | 18.8 g/dL |
| Heparin | 10 U/mL | 37.6 U/mL |
| Imipenem / Cilastatin | 100 mg/L | 376 mg/L |
| Vancomycin | 100 mg/L | 376 mg/L |
| Cefotaxime | 400 mg/L | 1504 mg/L |
| Dopamine | 500 mg/dL | 1880 mg/dL |
| CRP (C-reactive protein) | 60 mg/L | 225.6 mg/L |
| Norepinephrine | 670 µmol/L | 2519.2 µmol/L |
| Dobutamine | 11.2 mg/L | 42.1 mg/L |
| Furosemide | 59.9 mg/L | 225.2 mg/L |
| IL-6 (Interleukin-6) | 2000 pg/mL | 7520 pg/mL |
| sCD14 | 5 µg/mL | 18.8 µg/mL |
| LPS (Lipopolysaccharides) | 5 ng/mL | 18.8 ng/mL |

(*) Considering a standard mixture of 2.5 mL of whole blood in 6.9 mL of PAXgene solution.

## Supplementary Table 4. Analytical sensitivity and linearity sample preparation


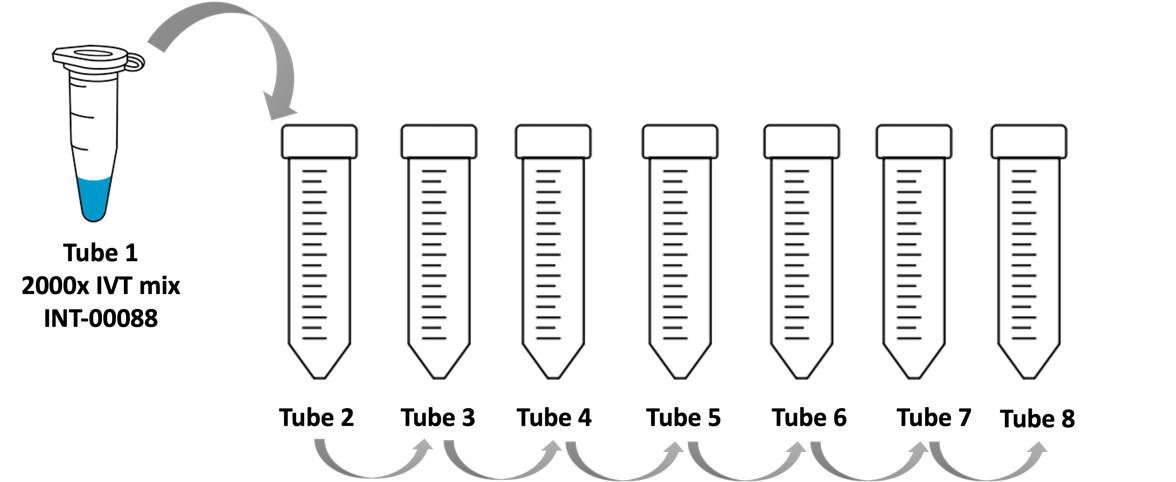


| **Scheme tube number** | **Concentration under testing** | **Volume of solution (mL)** | **Volume of PrimeStore (mL)** | **Total volume (mL)** |
| --- | --- | --- | --- | --- |
| 2 | 5E+09 cp/mL | 0.16 | 6.24 | 6.4 |
| 3 | 1E+09 cp/mL | 1.28 | 5.12 | 6.4 |
| 4 | 1E+08 cp/mL | 0.64 | 5.76 | 6.4 |
| 5 | 1E+07 cp/mL | 1 | 9 | 10 |
| 6 | 1E+06 cp/mL | 5.5 | 49.5 | 55 |
| 7 | 5E+05 cp/mL | 21 | 21 | 42 |
| 8 | 1E+05 cp/mL | 8 | 32 | 40 |

## Supplementary Table 5. Analytical sensitivity

| **Gene** | **Total Tests (N)** | **Gene Amplification** | | | | | |
| --- | --- | --- | --- | --- | --- | --- | --- |
|  |  | **1x10^6^ cp/mL**  **% (Valid/Total replicates)** | | **5x10^5^ cp/mL**  **% (Valid/Total replicates)** | | **1x10^5^ cp/mL**  **% (Valid/Total replicates)** |  |
| ANKRD22 | 60 | 100 (20/20) | 100 (20/20) | | 95 (19/20) | | |
| ARG1 | 60 | 100 (20/20) | 100 (20/20) | | 95 (19/20) | | |
| BATF | 60 | 100 (20/20) | 100 (20/20) | | 95 (19/20) | | |
| C3AR1 | 60 | 100 (20/20) | 100 (20/20) | | 95 (19/20) | | |
| CD163 | 60 | 100 (20/20) | 100 (20/20) | | 95 (19/20) | | |
| CEACAM1 | 60 | 100 (20/20) | 100 (20/20) | | 95 (19/20) | | |
| CLEC5A | 60 | 100 (20/20) | 100 (20/20) | | 95 (19/20) | | |
| CTSL1 | 60 | 100 (20/20) | 100 (20/20) | | 100 (20/20) | | |
| DEFA4 | 60 | 100 (20/20) | 100 (20/20) | | 95 (19/20) | | |
| ERCC17 | 60 | 100 (20/20) | 100 (20/20) | | 100 (20/20) | | |
| ERCC59 | 60 | 100 (20/20) | 100 (20/20) | | 100 (20/20) | | |
| HERC5 | 60 | 100 (20/20) | 100 (20/20) | | 95 (19/20) | | |
| HLA-DMB | 60 | 100 (20/20) | 100 (20/20) | | 90 (18/20) | | |
| IFI27 | 60 | 100 (20/20) | 100 (20/20) | | 90 (18/20) | | |
| IFI44 | 60 | 100 (20/20) | 100 (20/20) | | 95 (19/20) | | |
| IFI44L | 60 | 100 (20/20) | 100 (20/20) | | 90 (18/20) | | |
| IL18R1 | 60 | 100 (20/20) | 100 (20/20) | | 95 (19/20) | | |
| IL1R2 | 60 | 100 (20/20) | 90 (18/20) | | 40 (8/20) | | |
| ISG15 | 60 | 100 (20/20) | 100 (20/20) | | 95 (19/20) | | |
| JUP | 60 | 100 (20/20) | 100 (20/20) | | 95 (19/20) | | |
| KCNJ2 | 60 | 100 (20/20) | 100 (20/20) | | 95 (19/20) | | |
| KPNA6 | 60 | 100 (20/20) | 100 (20/20) | | 100 (20/20) | | |
| LY86 | 60 | 100 (20/20) | 100 (20/20) | | 95 (19/20) | | |
| OASL | 60 | 100 (20/20) | 100 (20/20) | | 95 (19/20) | | |
| OLFM4 | 60 | 100 (20/20) | 100 (20/20) | | 95 (19/20) | | |
| PSMB9 | 60 | 100 (20/20) | 100 (20/20) | | 95 (19/20) | | |
| RREB1 | 60 | 100 (20/20) | 100 (20/20) | | 95 (19/20) | | |
| RSAD2 | 60 | 100 (20/20) | 100 (20/20) | | 95 (19/20) | | |
| S100A12 | 60 | 100 (20/20) | 100 (20/20) | | 90 (18/20) | | |
| TDRD9 | 60 | 100 (20/20) | 100 (20/20) | | 90 (18/20) | | |
| TGFBI | 60 | 100 (20/20) | 100 (20/20) | | 95 (19/20) | | |
| XAF1 | 60 | 100 (20/20) | 100 (20/20) | | 80 (16/20) | | |
| YWHAB | 60 | 100 (20/20) | 100 (20/20) | | 95 (19/20) | | |
| ZDHHC19 | 60 | 100 (20/20) | 100 (20/20) | | 95 (19/20) | | |

## Supplementary Table 6: Linearity

| **Linearity 1x10^6^ copies/mL to 1x10^9^ copies/mL** | | | | |
| --- | --- | --- | --- | --- |
| **Gene** | **Slope** | **Intercept** | **Pearson** | **ADL 5%** |
| ANKRD22 | -1.65 | 27.61 | 0.989 | Pass |
| ARG1 | -1.91 | 31.19 | 0.985 | Pass |
| BATF | -2.03 | 35.08 | 0.988 | Pass |
| C3AR1 | -1.88 | 31.14 | 0.993 | Pass |
| CD163 | -1.61 | 27.66 | 0.993 | Pass |
| CEACAM1 | -2.10 | 35.61 | 0.981 | Pass |
| CLEC5A | -1.78 | 30.00 | 0.979 | Pass |
| CTSL1 | -2.06 | 33.66 | 0.997 | Pass |
| DEFA4 | -1.85 | 30.31 | 0.996 | Pass |
| HERC5 | -1.97 | 32.63 | 0.982 | Pass |
| HLA-DMB | -2.36 | 39.00 | 0.995 | Pass |
| IFI27 | -1.84 | 32.00 | 0.980 | Pass |
| IFI44 | -1.85 | 30.30 | 0.994 | Pass |
| IFI44L | -2.55 | 41.69 | 0.996 | Pass |
| IL18R1 | -1.85 | 30.33 | 0.993 | Pass |
| IL1R2 | -2.95 | 40.95 | 0.961 | Pass |
| ISG15 | -1.74 | 30.82 | 0.996 | Pass |
| JUP | -2.03 | 34.26 | 0.990 | Pass |
| KCNJ2 | -1.84 | 30.55 | 0.996 | Pass |
| KPNA6 | -1.89 | 31.37 | 0.995 | Pass |
| LY86 | -1.73 | 28.66 | 0.995 | Pass |
| OASL | -1.57 | 27.59 | 0.982 | Pass |
| OLFM4 | -2.41 | 37.80 | 0.991 | Pass |
| PSMB9 | -2.06 | 34.37 | 0.989 | Pass |
| RREB1 | -2.15 | 34.02 | 0.991 | Pass |
| RSAD2 | -1.84 | 33.40 | 0.961 | Pass |
| S100A12 | -2.52 | 40.38 | 0.995 | Pass |
| TDRD9 | -2.06 | 32.94 | 0.977 | Pass |
| TGFBI | -2.10 | 35.75 | 0.989 | Pass |
| XAF1 | -2.20 | 35.77 | 0.986 | Pass |
| YWHAB | -2.95 | 43.84 | 0.943 | Pass |
| ZDHHC19 | -2.06 | 34.41 | 0.974 | Pass |

ADL, average deviation from linearity

## Supplementary Table 7: Endogenous and exogenous interferences

| **Reagent** | **Concen-**  **tration** | **Panel** | **N** | **Average TriVerity Score** | | | **SD** | | | **Delta**  **Control vs. Spike-In** | | |
| --- | --- | --- | --- | --- | --- | --- | --- | --- | --- | --- | --- | --- |
|  |  |  |  | Bacterial | Viral | Illness Severity | Bacterial | Viral | Illness Severity | Bacterial | Viral | Illness Severity |
| No spike-in | N/A | B | 4 | 46.00 | 9.50 | 46.50 | 0.82 | 2.65 | 1.29 | N/A | N/A | N/A |
|  |  | C | 4 | 1.00 | 49.25 | 6.75 | 0.00 | 0.50 | 1.26 | N/A | N/A | N/A |
| NaOH | N/A | B | 4 | 46.50 | 7.50 | 47.75 | 1.29 | 4.12 | 0.50 | 0.50 | -2.00 | 1.25 |
|  |  | C | 4 | 0.75 | 49.00 | 6.75 | 0.50 | 0.00 | 0.96 | -0.25 | -0.25 | 0.00 |
| Serum | N/A | B | 4 | 46.00 | 10.50 | 46.50 | 0.82 | 3.32 | 1.00 | 0.00 | 1.00 | 0.00 |
|  |  | C | 4 | 1.00 | 49.25 | 7.25 | 0.00 | 0.50 | 1.26 | 0.00 | 0.00 | 0.50 |
| Water | N/A | B | 8 | 45.75 | 9.88 | 47.00 | 0.71 | 2.10 | 0.53 | -0.25 | 0.38 | 0.50 |
|  |  | C | 8 | 1.00 | 49.13 | 6.38 | 0.00 | 0.35 | 1.69 | 0.00 | -0.13 | -0.38 |
| Bilirubin | 400 mg/L | B | 4 | 46.25 | 8.50 | 47.00 | 0.96 | 2.38 | 1.41 | 0.25 | -1.00 | 0.50 |
|  |  | C | 4 | 1.00 | 49.25 | 7.00 | 0.00 | 0.50 | 1.83 | 0.00 | 0.00 | 0.25 |
| Hemoglobin | 10 g/L | B | 4 | 46.50 | 8.00 | 47.00 | 1.29 | 2.94 | 1.15 | 0.50 | -1.50 | 0.50 |
|  |  | C | 4 | 0.75 | 49.25 | 5.75 | 0.50 | 0.50 | 0.50 | -0.25 | 0.00 | -1.00 |
| Rheumatoid Factor | 45 U/mL | B | 4 | 46.75 | 8.00 | 46.50 | 0.96 | 1.63 | 0.58 | 0.75 | -1.50 | 0.00 |
|  |  | C | 4 | 1.00 | 49.00 | 6.25 | 0.00 | 0.00 | 0.96 | 0.00 | -0.25 | -0.50 |
| Triglycerides | 2000 mg/dL | B | 4 | 46.50 | 8.00 | 47.75 | 0.58 | 1.41 | 0.50 | 0.50 | -1.50 | 1.25 |
|  |  | C | 4 | 1.25 | 49.00 | 7.00 | 0.50 | 0.00 | 1.41 | 0.25 | -0.25 | 0.25 |
| Albumin | 5 g/dL | B | 4 | 46.50 | 9.50 | 46.25 | 1.29 | 4.20 | 1.71 | 0.50 | 0.00 | -0.25 |
|  |  | C | 4 | 1.00 | 49.25 | 7.25 | 0.00 | 0.50 | 2.50 | 0.00 | 0.00 | 0.50 |
| Heparin | 10 U/mL | B | 4 | 46.75 | 7.75 | 45.75 | 0.96 | 1.50 | 0.96 | 0.75 | -1.75 | -0.75 |
|  |  | C | 4 | 1.50 | 49.00 | 8.50 | 0.58 | 0.00 | 1.73 | 0.50 | -0.25 | 1.75 |
| Imipenem/ Cilastatin | 100 mg/L | B | 4 | 47.00 | 7.00 | 46.00 | 0.82 | 2.16 | 1.41 | 1.00 | -2.50 | -0.50 |
|  |  | C | 4 | 2.00 | 48.75 | 7.50 | 1.41 | 0.50 | 1.91 | 1.00 | -0.50 | 0.75 |
| Vancomycin | 100 mg/L | B | 4 | 46.75 | 8.00 | 46.25 | 1.50 | 4.32 | 0.50 | 0.75 | -1.50 | -0.25 |
|  |  | C | 4 | 0.75 | 49.25 | 6.25 | 0.50 | 0.50 | 2.87 | -0.25 | 0.00 | -0.50 |
| Cefotaxime | 400 mg/L | B | 4 | 47.50 | 6.75 | 47.75 | 1.00 | 2.22 | 0.50 | 1.50 | -2.75 | 1.25 |
|  |  | C | 4 | 1.00 | 49.00 | 6.25 | 0.00 | 0.00 | 0.96 | 0.00 | -0.25 | -0.50 |
| Dopamine | 500 mg/dL | B | 4 | 46.75 | 7.75 | 47.00 | 0.96 | 1.50 | 0.82 | 0.75 | -1.75 | 0.50 |
|  |  | C | 4 | 1.50 | 49.25 | 7.75 | 1.00 | 0.50 | 0.96 | 0.50 | 0.00 | 1.00 |
| CRP | 60 mg/L | B | 4 | 46.50 | 9.00 | 47.00 | 0.58 | 1.63 | 0.82 | 0.50 | -0.50 | 0.50 |
|  |  | C | 4 | 1.50 | 49.00 | 6.50 | 1.00 | 0.82 | 4.04 | 0.50 | -0.25 | -0.25 |
| Norepinephrine | 670 µmol/L | B | 4 | 46.25 | 8.50 | 47.50 | 0.50 | 0.58 | 0.58 | 0.25 | -1.00 | 1.00 |
|  |  | C | 4 | 1.00 | 49.25 | 5.50 | 0.00 | 0.50 | 0.58 | 0.00 | 0.00 | -1.25 |
| Dobutamine | 11.2 mg/L | B | 4 | 47.00 | 7.00 | 47.25 | 0.00 | 0.82 | 0.96 | 1.00 | -2.50 | 0.75 |
|  |  | C | 4 | 0.75 | 49.25 | 6.75 | 0.50 | 0.50 | 1.71 | -0.25 | 0.00 | 0.00 |
| Furosemide | 59.9 mg/L | B | 4 | 46.50 | 9.25 | 46.25 | 0.58 | 2.87 | 2.22 | 0.50 | -0.25 | -0.25 |
|  |  | C | 4 | 1.00 | 49.00 | 6.50 | 0.00 | 0.00 | 1.00 | 0.00 | -0.25 | -0.25 |
| IL-6 | 2000 pg/mL | B | 4 | 48.00 | 5.50 | 48.50 | 0.82 | 1.91 | 0.58 | 2.00 | -4.00 | 2.00 |
|  |  | C | 4 | 0.75 | 49.25 | 6.75 | 0.50 | 0.50 | 1.50 | -0.25 | 0.00 | 0.00 |
| sCD14 | 5 µg/mL | B | 4 | 46.50 | 8.50 | 46.75 | 0.58 | 1.29 | 0.50 | 0.50 | -1.00 | 0.25 |
|  |  | C | 4 | 1.00 | 49.00 | 6.00 | 0.00 | 0.00 | 1.63 | 0.00 | -0.25 | -0.75 |
| LPS | 5 ng/mL | B | 4 | 46.00 | 9.50 | 47.25 | 0.82 | 2.89 | 0.50 | 0.00 | 0.00 | 0.75 |
|  |  | C | 4 | 1.25 | 49.00 | 8.50 | 0.50 | 0.00 | 2.38 | 0.25 | -0.25 | 1.75 |

SD, standard deviation

N/A, not applicable

## Supplementary Table 8. Cross reactivity with genomic DNA

|  |  |  | **Average score**  **(SD)** | | |  | | **Delta control vs. gDNA (Score)** | | |  | | **Interpretation band**  **concordance** | | | |
| --- | --- | --- | --- | --- | --- | --- | --- | --- | --- | --- | --- | --- | --- | --- | --- | --- |
|  |  |  | **Bacterial** | **Viral** | **Severity** | |  | **Bacterial** | **Viral** | **Severity** | |  | **Bacterial** | **Viral** | **Severity** |  |
| **Control** |  | Contrived Sample B | 46.83  (0.75) | 8.50  (2.35) | 47.17  (1.17) | |  | N/A | N/A | N/A | |  | N/A | N/A | N/A |  |
|  |  | Contrived Sample C | 0.67 (0.52) | 49.67  (0.52) | 5.33  (1.21) | |  | N/A | N/A | N/A | |  | N/A | N/A | N/A |  |
| **gDNA** |  | Contrived Sample B | 46.00 (0.89) | 9.00  (2.10) | 45.33  (1.75) | |  | 0.83 | 0.50 | 1.83 | |  | 0 | 1 | 0 |  |
|  |  | Contrived Sample C | 1  (0) | 49.00 (0) | 5  (1.26) | |  | 0.33 | 0.66 | 0.33 | |  | 1 | 0 | 0 |  |

N/A, not applicable

## Supplementary Table 9. TriVerity Cartridge stability

|  | | | | **Average** | | | **Delta to control** | | | **SD (<5.5)** | | |
| --- | --- | --- | --- | --- | --- | --- | --- | --- | --- | --- | --- | --- |
| **Time point** | **Days** | **Temp (ºC)** | **Panel member** | **Bacterial** | **Viral** | **Severity** | **Bacterial** | **Viral** | **Severity** | **Bacterial** | **Viral** | **Severity** |
| 0 | 0 | RT | B | 47.00 | 7.83 | 48.00 | N/A | N/A | N/A | 0.00 | 0.98 | 0.89 |
|  |  |  | C | 1.00 | 49.67 | 7.33 | N/A | N/A | N/A | 0.00 | 0.52 | 1.51 |
| 3M | 100 | 15 | B | 46.33 | 9.17 | 48.67 | -0.67 | 1.33 | 0.67 | 0.82 | 3.37 | 0.52 |
|  |  |  | C | 0.83 | 49.50 | 6.17 | -0.17 | -0.17 | -1.17 | 0.41 | 0.55 | 1.17 |
|  | 100 | 30 | B | 46.00 | 10.83 | 48.17 | -1.00 | 3.00 | 0.17 | 0.89 | 3.19 | 0.75 |
|  |  |  | C | 1.00 | 49.17 | 5.00 | 0.00 | -0.50 | -2.33 | 0.00 | 0.41 | 0.63 |
|  | 62 | 37 | B | 45.83 | 9.33 | 47.17 | -1.17 | 1.50 | -0.83 | 1.17 | 2.94 | 0.98 |
|  |  |  | C | 1.00 | 49.17 | 4.50 | 0.00 | -0.50 | -2.83 | 0.00 | 0.41 | 1.05 |
| 6M | 192 | 15 | B | 46.00 | 9.50 | 48.50 | -1.00 | 1.67 | 0.50 | 1.10 | 2.35 | 0.55 |
|  |  |  | C | 1.00 | 49.50 | 6.50 | 0.00 | -0.17 | -0.83 | 0.00 | 0.55 | 1.05 |
|  | 192 | 30 | B | 45.50 | 10.67 | 47.17 | -1.50 | 2.83 | -0.83 | 1.87 | 3.88 | 2.14 |
|  |  |  | C | 1.00 | 49.17 | 4.67 | 0.00 | -0.50 | -2.67 | 0.00 | 0.41 | 1.51 |
|  | 119 | 37 | B | 47.17 | 7.17 | 48.67 | 0.17 | -0.67 | 0.67 | 1.17 | 2.64 | 0.52 |
|  |  |  | C | 1.00 | 49.00 | 6.83 | 0.00 | -0.67 | -0.50 | 0.00 | 0.00 | 1.33 |
| 9M | 283 | 15 | B | 46.17 | 9.50 | 48.50 | -0.83 | 1.67 | 0.50 | 1.17 | 3.78 | 0.55 |
|  |  |  | C | 0.83 | 49.17 | 6.67 | -0.17 | -0.50 | -0.67 | 0.41 | 0.41 | 1.03 |
|  | 283 | 30 | B | 46.83 | 7.00 | 48.17 | -0.17 | -0.83 | 0.17 | 0.98 | 1.67 | 0.75 |
|  |  |  | C | 1.33 | 48.67 | 6.17 | 0.33 | -1.00 | -1.17 | 0.52 | 0.52 | 1.72 |
|  | 175 | 37 | B | 47.00 | 7.17 | 48.17 | 0.00 | -0.67 | 0.17 | 0.63 | 2.23 | 0.75 |
|  |  |  | C | 1.00 | 49.00 | 7.83 | 0.00 | -0.67 | 0.50 | 0.00 | 0.00 | 1.17 |
| 12M | 231 | 37 | B | 47.33 | 6.17 | 48.40 | 0.33 | -1.67 | 0.40 | 0.52 | 1.83 | 0.55 |
|  |  |  | C | 1.50 | 49.17 | 8.83 | 0.50 | -0.50 | 1.50 | 0.55 | 0.41 | 2.32 |

N/A, Not Applicable

## Supplementary Table 10. TriVerity Fresh-Frozen Equivalency Demonstrated using Score Mean Shift

|  | **Mean Shift in TriVerity Scores (95% CI) (N=60)** | | | |
| --- | --- | --- | --- | --- |
| **Score** | **Time of Blood Collection (T_0_)**  **vs. 24 +/- 2 hrs at RT (T_1_)** | **p-value** | **Time of Blood Collection (T_0_)**  **vs. 24 +/- 2 hrs** **at -80**°**C (T_2_)** | **p-value** |
| Bacterial | -1.6 (-3.5, -0.5) | 0.02 | - 2.1 (-4.5, -1.0) | 0.002 |
| Viral | 1.65 (0.5, 2.5) | 0.005 | 0.83 (0.0, 2.5) | 0.09 |
| Severity | 0.98 (-0.5, 3.0) | 0.21 | 1.23 (-0.5, 3.0) \ | 0.13 |

 CI, confidence interval
 RT, room temperature

## Supplementary Table 11: Intra-and inter-platform concordance of the Myrna Instrument and NanoString nCounter for TriVerity Bacterial and Viral scores.

| **TriVerity Score** | **Myrna**  **vs.**  **Myrna**  **(N^1^ = 278)** | **Nanostring**  **vs.** **Nanostring**  **(N^1^ = 524)** | **Myrna**  **vs.**  **Nanostring**  **(N^1^ = 657)** |
| --- | --- | --- | --- |
| Bacterial score^2^ | 0.95 | 0.989 | 0.913 |
| Bacterial band concordance [%]^3^ | 98 | 100 | 96 |
| Viral score^2^ | 0.97 | 0.97 | 0.934 |
| Viral band concordance [%]^3^ | 99 | 98 | 978 |
| Severity score^2^ | 0.92 | 0.98 | 0.92 |
| Severity band concordance [%]^3^ | 99 | 100 | 96 |

^1^ N is the number of distinct matched pairs of samples

^2^ Pearson coefficient of correlation was used to compare the results obtained between the two instruments

^3^ Band concordance is the percentage of replicate samples assigned to the same or neighboring band by the test
